# Transcriptional, Epigenetic and Pharmacological Control of JAK/STAT Pathway in NK Cells

**DOI:** 10.3389/fimmu.2019.02456

**Published:** 2019-10-17

**Authors:** Gianluca Scarno, Giuseppe Pietropaolo, Chiara Di Censo, Massimo Gadina, Angela Santoni, Giuseppe Sciumè

**Affiliations:** ^1^Department of Molecular Medicine, Laboratory Affiliated to Istituto Pasteur Italia—Fondazione Cenci Bolognetti, Sapienza University of Rome, Rome, Italy; ^2^Translational Immunology Section, Office of Science Technology (OST), National Institute of Arthritis and Musculoskeletal and Skin Diseases, NIH, Bethesda, MD, United States; ^3^IRCCS Neuromed, Pozzilli, Italy

**Keywords:** NK cells, innate lymphoid cells, JAK, STAT, cytokine, transcriptome, transcription factor

## Abstract

Differentiation of Natural Killer (NK) cells is a stepwise process having its origin in the bone marrow and proceeding in the periphery, where these cells follow organ specific trajectories. Several soluble factors and cytokines regulate the distinct stages of NK cell differentiation, and ultimately, their functional properties. Cytokines activating the Janus kinases (JAKs) and members of the signal transducer and activator of transcription (STAT) pathway control distinct aspects of NK cell biology, ranging from development, terminal differentiation, activation, and generation of cells with adaptive properties. Here, we discuss how the recent advances of next generation sequencing (NGS) technology have led to unravel novel molecular aspects of gene regulation, with the aim to provide genomic views of how STATs regulate transcriptional and epigenetic features of NK cells during the different functional stages.

## Introduction

Natural Killer (NK) cells are the founding members of the ILC family and represent the innate counterpart of cytotoxic T lymphocytes (1, 2). Like CD8^+^ T cells, NK cells are able to kill infected or transformed cells in a perforin and granzyme dependent manner, as well, these cells are able to mount a rapid type-1 response by releasing the eponymous cytokine, interferon (IFN)-γ (3, 4). NK cells share the ability to produce type-1 cytokines with a distinct “helper” prototypical innate subset, termed ILC1 (5, 6). NK cells differ from ILC1 for their cytotoxic abilities, for a higher propension to circulate in the bloodstream and for the expression of lineage defining transcription factors (LDTFs) (7–9). In this regard, both NK cells and ILC1 are regulated by transcription factors (TFs) of the T-box family; however, while Eomes is expressed and required only by NK cells, T-bet (encoded by *Tbx21*) is expressed by both prototypical subsets (10–14). Expression of T-bet is fundamental for the generation of ILC1, and it also has non-redundant roles in regulating NK cell turnover, effector functions and egression from bone marrow (10, 11, 15).

Cytokines and other soluble factors regulate several aspects of NK cell biology, acting through signal-dependent TFs (SDTFs). In particular, cytokines activating the Janus kinases (JAKs) and members of the signal transducer and activator of transcription (STAT) pathway control NK cell development, terminal differentiation, acquisition of effector phenotype up to generation of cells with adaptive features able to provide secondary responses (16, 17). Mammalian genomes contain four genes encoding for JAKs, namely JAK1, JAK2, JAK3, and TYK2; and seven genes for STATs, STAT1-4, STAT5A, STAT5B, and STAT6 (18, 19). Activation of the JAK tyrosine kinases occurs upon receptor engagement, and the juxtaposition of JAKs and STATs allows, after phosphorylation, STAT dimers to dissociate from the membrane complex and to migrate into the nucleus, where they bind specific DNA-motifs modulating gene expression (20).

The role of the JAK/STAT dependent signals on NK cells and other ILCs has been discussed in recent reviews (16, 17, 21); herein, we focus on the molecular mechanisms underlying NK cell differentiation in physiological and pathological contexts. We discuss how the advances of next generation sequencing (NGS) technology and the establishment of novel mouse models have led to a better definition of the genes regulated by STATs, and their transcriptional and epigenetic control of NK cells during differentiation and host defense. Finally, we provide an overview of the JAK inhibitors currently approved for the treatment of immune-mediated disorders and their possible implication on NK cells.

## STAT5 as a Central Node for Development, Identity and Homeostasis of NK Cells

The bone marrow is the main site for NK cell and ILC development in the adult, containing distinct progenitors and precursors able to give rise to cells having different fates (22, 23). Differentiation proceeds with a pool of circulating progenitors which move to the periphery, where NK cells and other ILCs follow organ specific trajectories and acquire distinct effector functions (24). In the current model, NK cells have a dedicated pathway of differentiation comprising a pool of committed NK cell precursors (NKps) (25–27). Their differentiation follows a stepwise process encompassing distinct developmental and/or functional stages, discriminated through the expression of CD27 and CD11b levels in mice and CD56 and CD16 in humans [redefined recently by single cell RNA-seq approach (28, 29)].

The cytokines IL-7 and IL-15 are critical for lymphoid development by transmitting their signals through the common IL-2 γ-chain receptor (CD132) and by activating JAK3, JAK1, and STAT5 (30). Deletion of *Jak3* in mice is associated with reduced numbers of lymphoid and ILC precursors, in contrast to an accumulation of NKp (31). This evidence is in line with previous findings demonstrating that IL-15 was required for the NKp to proceed toward the next maturation stages (25). Similarly, mice carrying conditional deletion of *Jak1* in *Ncr1*-expressing cells (*Jak1*^*fl*/*fl*^ *Ncr1Cre*) show profound defects in NK cell differentiation and homeostasis; *Jak2* deletion, instead, does not affect NK cell development and survival (32).

JAK3 and JAK1 mainly activate STAT5, which represents a key multi-lineage TF (MLTF) controlling development of both adaptive and innate lymphocytes (33, 34). Ablation of the entire *Stat5* locus, comprising both *Stat5a* and *Stat5b*, results in a high perinatal lethality, due to the pleiotropic role of this TF; however, the few viable *Stat5*^−/−^ mice show absence of NK cells (35). Conditional deletion of *Stat5* in Ncr1-expressing cells allows to eliminate the confounding effects related to lymphopenia and inflammation observed in mice carrying germline ablation; in these settings, both development and survival of NK cells remain highly impaired (36).

Due to the massive effect of STAT5 deletion on NK cells, our understanding of how this SDTF works at the molecular level has remained elusive; the use of mice bearing only one allele of STAT5 has helped to clarify this aspect. Between the two paralogs, *Stat5b* is more expressed than *Stat5a* in innate and adaptive lymphocytes, and its deletion has broad effects on NK cell differentiation (37–39). Transcriptomic analyses performed on NK cells retaining only one *Stat5* allele (*Stat5a*^−/−^*Stat5b*^+/−^) have shed light on the homeostatic impact of this TF on NK cells, which consists on regulation of over 400 genes (39). The residual NK cells present in these mice show a developmental block associated with an accumulation of CD11b^low^ cells, and a drastic decrease of the expression of the anti-apoptotic gene, *Bcl2*. Along with defects in development and survival, STAT5 sustains the expression of most of the genes (52 out of 76) defining NK cell identity, including NKG2D, perforin and granzymes, and the LDTF T-bet (39). These findings have helped to discriminate between the instructive role of STAT5 during NK cell differentiation and its permissive function in regulating survival.

**Figure 1:**
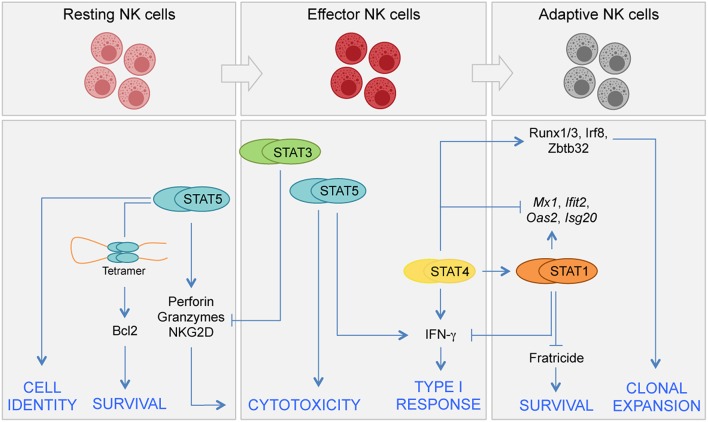
Distinct requirements for STATs in NK cell differentiation. JAK/STAT signals control several aspects of NK cell biology, including development, terminal differentiation, acquisition of effector functions, and generation of adaptive NK cells. NK cell development begins in the bone marrow from committed precursors and it is driven by signals inducing STAT5 activation. In homeostatic conditions STAT5 sustains NK cell survival by direct regulation of Bcl2 expression. STAT5 is also required for terminal differentiation and acquisition of NK cell identity. STAT4 and STAT1 have both specific and shared roles during viral infection. STAT4 controls a network of TFs required for clonal expansion of NK cells during proliferation. STAT4 and STAT1 compete at genomic level for the expression of IFN-γ and other genes. STAT3 has a role in restraining NK cell effector functions by inhibiting perforin, granzyme B, and NKG2D expression.

Upon activation, STAT5 can form dimers but also tetramers having distinct ability to interact with DNA-regulatory elements (40, 41). While STAT5 dimers bind to canonical GAS (IFN-γ activation site, TTCN3GAA) motif, STAT5 tetramers bind to divergent motifs having an optimal spacing of 2–27 base pairs between GAS and GAS-like sequences. The relative importance of STAT5 dimers vs. tetramers in NK cells has been evaluated by the generation of a mouse model carrying genes encoding for tetramer defective mutant STAT5 proteins (40, 42). In these mice, the impaired STAT5 binding to the *Bcl2* locus, and the consequent lower mRNA and protein expression, leads to a more rapid cell death of NK cells compared to wild type cells (40). Interestingly, transgenic expression of Bcl2 is able to rescue the effect of *Stat5* deficiency on the homeostatic pool of NK cells (43). These “*Bcl2*-rescued” NK cells undergo a functional switch from tumor-suppressive to tumor-promoting cells, since loss of STAT5 determines upregulation of the pro-angiogenic factor VEGFA, which sustains tumor growth (43). Thus, while STAT5 represents a central node in NK cell development, acquisition of cell identity, and homeostasis (Figure 1), the involvement of other STATs in regulating these processes appears limited. Of note, type I IFNs and STAT1 can have distinct indirect effects on NK cell homeostasis: including the regulation of MHC class I expression (44), as well as the regulation of the production and trans-presentation of IL-15 on accessory cells (45–47).

## Multiple STATs Underlie Effector Functions of NK Cells

Effector functions of NK cells depend both on cytokines and on a complex equilibrium between activating and inhibitory receptors, which bind molecules present on healthy and stressed cells including MHC class I and adhesion molecules (48–52). The ability of NK cells to sense environmental changes and rapidly release their effector potentials is favored by a primed epigenetic and transcriptional state, leading to high basal expression of cytokine receptors, LDTFs, and SDTFs, including STATs (53). Although distinct STATs can be easily linked to particular effector and helper functions, it is now clear that activation of NK cells, like the other ILCs, can be influenced by complementary actions of multiple STATs (54–56).

Acting downstream of IL-12, STAT4 is necessary to mount a proper innate response against pathogens by actively regulating NK effector functions, including both IFN-γ production and cytotoxic response (57). The global impact of STAT4 in NK cell activation has been recently tackled using transcriptomic and epigenetic approaches (58, 59). Upon cytokine stimulation, over 300 differentially expressed genes are bound by STAT4 within or in proximity of the locus (59). Along with direct regulation of key effector genes, STAT4 controls the expression of several TFs required for a proper antiviral response, including *Zbtb32, Runx1, Runx3*, and *Irf8*. At molecular level, STAT4 binds to the promoter and intergenic regions of the gene locus of these TFs, leading to an increase of the permissiveness of the transcription through modification of the chromatin state, via trimethylation of histone H3 lysine 4 (59). Mouse models carrying selective deletion of these TFs have helped to unravel their impact on the cell cycle program of NK cells during viral infection. The effects of Zbtb32 on the proliferative burst and protective ability of NK cells are mediated by antagonizing the anti-proliferative effects of the TF Blimp-1 (encoded by *Prdm1*) (60); Irf8, instead, regulates proliferation acting upstream of Zbtb32 (61). During the course of viral infection, the expression of STAT4 and STAT1 follows an opposite fate. Indeed, while STAT4 expression is down-regulated, STAT1 results progressively up-regulated (58, 62). This differential expression pattern affects the signaling downstream of type I IFNs, which mainly activates STAT4 in the early phases of infection and STAT1 in later phases. The increased levels of STAT1 cause a displacement of STAT4 from type I IFN receptors, this switch induces a STAT1 dependent down-regulation of IFN-γ production in NK cells (62).

The role of STAT3 on NK cells has been dissected by employing distinct mouse models, showing differential effects whether deletion of *Stat3* gene occurs before or after NK cell development (63, 64). When *Stat3*^*fl*/*fl*^ mice are crossed with *Tie2-Cre* mice, the effects of *Stat3* deletion extend to the whole hematopoietic compartment. In these settings, NK cells show a decreased expression of NKG2D and impaired effector functions (63). In line with these findings, NK cells from subjects with dominant-negative STAT3 mutations show an impaired expression of NKG2D both at steady state and after cytokine stimulation (63). On the other hand, specific deletion of *Stat3* in differentiated NK cells, using *Ncr1iCre Stat3*^*fl*/*fl*^ mice, leads to an increased expression of DNAM-1, Perforin, and Granzyme B, and enhanced anti-tumor activity, as the result of the possible repressive functions of STAT3 on these cells (64). Considering these conflicting findings, genome-wide studies aimed at dissecting the transcriptomic impact of *Stat3* deletion on NK cells would be particularly relevant to discriminate between the direct and indirect roles of this TF in regulating differentiation and effector functions.

Beyond the homeostatic requirement in sustaining the expression of NK effector molecules, cytokines activating STAT5 have been used to stimulate NK cell functions *in vitro*, for decades (65, 66). Genomic maps of STAT5 distribution obtained by ChIP-seq analysis have revealed a widespread DNA binding in untreated and IL-15-treated NK cells. However, the acute stimulation with IL-15 induces a redistribution of this TF to a new set of DNA regulatory elements. In these settings, STAT5 binding occurs on almost half of the differentially expressed genes. Gene set enrichment analysis (GSEA) have confirmed a positive enrichment for IL-2/STAT5 signaling in STAT5 bound genes (39). In contrast, unbound genes show a positive enrichment for downstream targets of the mTOR pathway, which has been shown to mediate IL-15-dependent functions in NK cells, including proliferation and terminal differentiation, by regulating CD122 (IL-2Rβ) and CD132 (IL-2Rγ) expression; as well as metabolism, and acquisition of cytolytic features (67, 68).

## Specific Roles for STATs During Formation of Adaptive NK Cells

In the context of viral infection, NK cells are able to provide secondary immune responses by following a differentiation path which leads to generation of long-lived cells, named “memory” or “adaptive” NK cells (69, 70). Changes of chromatin accessibility of NK cells have been tracked *in vivo* up to 35 days after MCMV infection, by ATAC-seq (58). This analysis has revealed that the epigenetic landscape of NK cells is highly dynamic, with the majority of chromatin remodeling occurring in the first 2 weeks. These modifications pave the way for a further acquisition of the transcriptional adaptive state, observed at later time points (58). Genomic maps of STAT4 and STAT1 distribution in cytokine-stimulated NK cells have shown a differential DNA occupancy, being STAT4 mainly localized at putative enhancer sites and STAT1 at promoter regions (58). In line with these results, during MCMV infection the chromatin accessibility of putative enhancer sites and promoters remains less accessible in NK cells deficient for STAT4 and STAT1, respectively. Moreover, due to the existing competitive effects between STAT4 and STAT1, deletion of *Stat1* in NK cells leads to an increased DNA accessibility of non-promoter regions; as well as, to an increased expression of selected STAT4 regulated genes, such as *Ifng*. Conversely, the expression of several STAT1 targets, including *Mx1, Ifit2, Oas2*, and *Isg20*, is upregulated in absence of *Stat4* (58).

The interplay between STATs and LDTFs is a further mechanism underlying acquisition of specific functions in innate lymphocytes, including the generation of the adaptive phenotype in NK cells. This is the case for the cross-regulation occurring between STATs and T-bet (39, 71, 72); while STAT5 induces T-bet expression in homeostatic conditions (39), STAT4 binds to *Tbx21* locus at a distal enhancer site and promotes T-bet expression during MCMV infection (72). T-bet and Eomes are both necessary for NK cell proliferation; however, the IL-12/STAT4/T-bet axis plays a non-redundant role for the maintenance of adaptive NK cells (72). We have discussed in the previous section the network of TFs induced by STAT4, namely Zbtb32, Runx1, Runx3, and Irf8, which are all necessary to enhance proliferation and clonal expansion of NK cells (59–61). As well, expression of STAT1 has a non-redundant role for survival, regulating a Bcl2-independent mechanism enabling NK cells to evade cell death after viral infection. In particular, type 1 IFNs and STAT1 are required to prevent a mechanism of NK cell mediated fratricide, occurring via NKG2D and perforin (73). Overall, these findings shed light on the complex network of TFs and molecules regulated by STATs, required for the acquisition of the adaptive traits by NK cells.

## Conclusion: Translational Relevance of Targeting the JAK/STAT Pathway in Inflammation and Cancer

Manipulation of cytokine signaling in NK cells and other ILCs is drawing a growing interest for the treatment of inflammatory diseases and cancer (74, 75). In particular, harnessing NK cell effector functions against cancer by interfering with cytokine signaling has led to promising results in several mouse models (76–79). In this context, the suppressor of cytokine signaling (SOCS) proteins are a class of natural regulators of the activity of STATs. The SOCS protein CIS (encoded by *Cish*) is at the top among the genes induced by STAT5 activation, and acts as a negative regulator of IL-15 signaling, preventing excessive activation (77). Targeting *Cish* has a huge impact in enhancing NK cell dependent tumor immunity in several mouse models (77, 79); thus, given its primary role in restraining NK cell functions, CIS represents a novel immune checkpoint for these cells.

On the other hand, several small molecules capable to inhibit JAKs enzymatic activity have been recently developed. At least five JAK inhibitors (also known as JAKinibs) are now approved by various regulatory agencies to treat immune-mediated disorders. These first-generation JAKinibs comprise ruxolitinib, a JAK1 and JAK2 inhibitor, approved for myeloproliferative malignancies; tofacitinib, a JAK1, JAK2, JAK3 inhibitor, approved for rheumatoid arthritis, psoriatic arthritis, and ulcerative colitis; baricitinib, a JAK1 and JAK2 inhibitor, approved for rheumatoid arthritis; peficitinib, a pan-JAK inhibitor approved (only in Japan) for the treatment of rheumatoid arthritis; and oclacitinib, a JAK1 and JAK2 inhibitor, approved for allergic dermatitis in dogs (80).

The impact of ruxolitinib in NK cell homeostasis and functions has been evaluated in humans in distinct contexts. Myelofibrosis patients undergoing ruxolitinib treatment show a defect in NK cell number and differentiation, as well as, impaired functions upon cytokine stimulation; these effects have been related to the increased rates of infection observed in these patients (81). Ruxolitinib also inhibits the generation and functions of cytokine-induced memory-like NK cells by interfering with both IL-15 and IL-12 signaling (82). Finally, Ruxolitinib administration can limit STAT1 activation in patients carrying STAT1 gain of function mutations. In these patients, the prolonged STAT1 activation leads to an impaired NK cell maturation and function, associated with lower STAT5 phosphorylation downstream of IL-15 stimulation, and with lower levels of perforin. These defects are partially reverted by ruxolitinib administration (83).

More selective agents have been developed and are currently being tested. These next-generation inhibitors may possess the advantage of a reduced toxicity. For example, selective targeting of JAK1 would spare interfering with many of JAK2-dependent cytokines involved in hematopoiesis, including Epo, Tpo, G-CSF, GM-CSF, IL-3, and IL-11. Conversely, their efficacy could also be limited. Recently, immunogenomic analysis of mice administered with several JAKinibs, including both first- and second-generation inhibitors, have highlighted the impact of blocking either one or both JAK1 and JAK3 on NK cell homeostasis. Moreover, the JAK1-specific inhibitor (PF-02384554) was more efficient than the JAK3-specific (PF-06651600) in blocking the secondary autocrine response to IFN-γ induced in IL-2 activated NK cells (84).

The optimal degree of JAK inhibition required for an individual cell type in any given tissue remains unknown. To this end, selective JAKinibs may be the key to provide new mechanistic insights in the modulation of the JAK/STAT pathway in NK cells. This approach could be more effective than the use of JAK-deficient mice, in which developmental defects can mask the functional relevance of each JAK. Finally, we are now aware that JAKinibs can affect the structure of the epigenome and preferentially impact genes with super-enhancer structure (85). Notably, several genes encoding for cytokines or their cognate receptors are located within loci with super-enhancer architecture. Therapeutically, it will be important to understand how these drugs, alone or in combination with other chemotherapeutic agents, can be used to effectively, and safely, regulate these critical loci and, in turn, immune as well as non-immune cells.

## Author Contributions

GSca, GP, CD, MG, AS and GSci wrote the manuscript. CD designed the figure and made the necessary edits. The final manuscript was a result of the joint efforts of all the authors.

### Conflict of Interest

The authors declare that the research was conducted in the absence of any commercial or financial relationships that could be construed as a potential conflict of interest.

## Abbreviations

ILC: innate lymphoid cell
IFN: interferon
LDTF: lineage defining transcription factor
SDTF: signal dependent transcription factor
NK: natural killer
STAT: Signal Transducer and Activator of Transcription.
